# Production of Bioactive Compounds from the Sulfated Polysaccharides Extracts of *Ulva lactuca*: Post-Extraction Enzymatic Hydrolysis Followed by Ion-Exchange Chromatographic Fractionation

**DOI:** 10.3390/molecules24112132

**Published:** 2019-06-05

**Authors:** Nihal Abou El Azm, Daisy Fleita, Dalia Rifaat, Eric Zadok Mpingirika, Asma Amleh, Mayyada M. H. El-Sayed

**Affiliations:** 1 Chemistry Department, American University in Cairo, AUC Avenue, New Cairo 11835, Egypt; Niho@aucegypt.edu (N.A.E.A.); Dfleita@aucegypt.edu (D.F.); drifaat@aucegypt.edu (D.R.); 2 Department of Biology, American University in Cairo, AUC Avenue, New Cairo 11835, Egypt; zadok@aucegypt.edu (E.Z.M.); Aamleh@aucegypt.edu (A.A.)

**Keywords:** algae, polysaccharides, enzymatic hydrolysis, ion exchange chromatography, antioxidant, antitumor activity

## Abstract

This paper describes a novel combined post-extraction process for obtaining bioactive compounds from the aqueous high molecular weight sulfated polysaccharides (SPs) extracts of the green algae, *Ulva lactuca*. After extracting the SPs, they were enzymatically hydrolyzed then the hydrolysate (V45) was fractionated into eight different molecular weight fractions (F1–F8) using ion exchange chromatography. Crude SPs together with V45 and (F1–F8) were examined for their carbohydrate, protein, and sulfate contents. In addition, their degree of polymerization (DP) was estimated and they were characterized by Fourier Transform Infrared Spectroscopy (FTIR). Fractions S1, F4, F5, and F8 showed promising antioxidant and antitumor activities in vitro. In particular, the remarkable antitumor activity of F5 on three types of cancer cell lines could be attributed to its comparable contents of protein, carbohydrate, and sulfate, in addition to its comparable contents of rhamnose and glucuronic acid, and the same for glucose and arabinose. F5 also possessed the highest Hill coefficient among the four promising fractions indicating a higher degree of cooperativity in ligand binding. Other influencing factors including DP, composition, and type of characteristic functional groups were also discussed. The implications of this work could potentially benefit the industries of food supplements and pharmaceuticals.

## 1. Introduction

In the past decades, marine seaweeds have gained much interest as wealthy resources of bioactive compounds. In particular, sulfated polysaccharides (SPs) extracted from algal species have shown various biological and physiological activities. 

*Ulva lactuca*, or as commonly known by “sea lettuce”, is an edible green seaweed from which the SPs, ulvans, are extracted. Not all ulvans are water soluble; the insoluble ones resemble cellulose [1]. Their molecular weight ranges from 189 to 8200 KDa and they are composed of units of mono or disaccharides such as rhamnose, xylose, and iduronic or glucuronic acid. The most abundant unit is ulvan biouronic acid with sulfate at C3 where the acid unit could be either glucuronic or iduronic acid [2].

Several studies reported the antioxidant activity of *Ulva lactuca* extracts [3,4,5,6,7,8,9]. In addition to their potent antioxidant activities, ulvans have shown promising anticancer activities via one or more of the following mechanisms; anti-metastasis, anti-angiogenesis, immunity modulation, apoptosis, and antioxidant activity [10]. SPs extracted from *Ulva intestinalis* and fractionated onto a silica–silica column demonstrated antioxidant in addition to immunomodulatory activities since they stimulated pro-inflammatory cytokines production [11]. Polysaccharides from the green algae, *Ulva fasciata*, exhibited anticancer effect on lymphoma cells (Dalton ascitic lymphoma), which were introduced to mice and showed promising results [12], however those from the same algae showed minimal inhibitory effects on gastric (MKN45) and intestinal (DLD) cancer cells [13]. Ulvans from French *Ulva lactuca* inhibited colon cancer cell growth as they induced a low cell reactivity to Ulex europaeus-1 lectins [14], while those of Egyptian origin were found to have potential in vivo and in vitro antitumor cytotoxicity effects on Ehrlich ascites carcinoma EAC-cells; as well as in vitro effect on hepatocellular carcinoma HepG2 and colon carcinoma HCT116 human cell lines [15]. Ulvans from Vietnamese *Ulva lactuca* were also shown to be cytotoxic to hepatocellular carcinoma, in addition to human breast and cervical cancer cell lines [16]. 

Studies were performed to understand the relation between the polysaccharides molecular weight and their antitumor activity, which is usually associated with antioxidant activity. Ulvans from *Ulva pertusa* were degraded with hydrogen peroxide and they showed better antioxidant activity than the non-degraded ones [3]. In addition, polysaccharide extracts of the red algae *Porphyridium cruentum*, were subjected to hermetical microwave to obtain different molecular weight fractions, the smallest of which yielded the highest antioxidant activities [17]. Furthermore, polysaccharides from *Saragasum pallidum* were extracted using different extraction techniques, then tested for their antioxidant and antitumor activities. Among the different extracts, it was found that the smallest molecular weight polysaccharides exhibited the best antitumor activity on A549 [18].

It was also reported that column purification enhances the antioxidant activity of the extracted SPs. Polysaccharides extracted from *Ganoderma atrum* and purified onto a gel filtration chromatography column manifested potent antioxidant activities and the activity of one of them was comparable to that of ascorbic acid [19]. Hot water polysaccharide extracts of *Gracilaria rubra* were purified onto anion exchange and gel filtration columns, and the purified fractions exhibited good antioxidant and immunostimulating activities [20]. In our previous work [21], we reported the enzymatic hydrolysis of the red algal extracts of *Pterocladia capillacea* using different enzymes. Each enzyme hydrolysate was applied onto an anion exchange column for the sake of purification and not fractionation. It was found that the viscozyme hydrolysate showed the highest antioxidant activities of more than 90%, before and after column purification.

**Figure 1:**
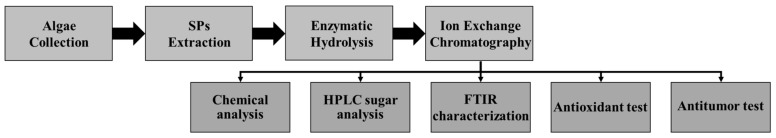
Illustration of the framework of this study.

As a follow up to our previous work where we showed that enzymatic hydrolysis improves the biological activity of crude extracts, we herein propose for the first time, to the best of our knowledge, a novel combined process of post-extraction hydrolysis followed by ion exchange column fractionation of algal SPs. The aim of this process is to produce different molecular weight fractions with different compositions and potentially various potent biological activities. By means of this facile process, products with enhanced biological activities could be obtained in a few number of steps and via a green approach with no solvent requirements. The study also looks into the parameters that might affect the antioxidant and antitumor activities of these fractions. The detailed framework of this study and the employed procedures are outlined in Figure 1. First, we extracted SPs from the green *Ulva lactuca* algae using the conventional water extraction technique. Then, we subjected the extracted SPs to an enzymatic hydrolysis treatment followed by anion exchange column fractionation. The different molecular weight column effluent fractions were chemically analyzed for their total carbohydrate, sulfate, protein, and monosaccharide content. They were also characterized using Fourier Transform Infrared Spectroscopy (FTIR). Finally, antioxidant and antitumor activities of the fractions were evaluated in order to determine the effect of molecular weight and composition on these activities. 

## 2. Results and Discussion

Here, we investigate the effect of the enzymatic hydrolysis and anion exchange chromatographic purification on the 2,2-diphenyl-1-picrylhydrazyl (DPPH) antioxidant and antitumor activities of the collected mother fraction (S1), the enzymatically-hydrolyzed fraction (V45), and the eight column effluent fractions (F1–F8). Chemical analysis and evaluation of the biological activities of these fractions will, thus, be employed to study this effect.

### 2.1. Chemical Analysis

The total protein, carbohydrate, and sulfate contents in each collected fraction are listed in Table 1, along with the degree of polymerization (DP).

Among the investigated fractions, S1 has the highest % carbohydrates (Table 1). With the enzymatic hydrolysis of S1 to produce V45, the carbohydrate and sulfate contents decreased at the expense of the protein content. This is probably because Viscozyme broke down the saccharide linkages and, as a result of centrifugation and decantation, some of the broken soluble sugars were removed. Among the eight column fractions, F4–F8 have the highest sulfate contents, with a corresponding degree of polymerization of 3–5. In addition, F4 has the highest protein content while F7 and F8 contain traces of proteins. It can also be deduced from the table that the algal extract (S1) contains comparable contents of carbohydrates and sulfates. The same holds true for each of fractions F4, F5, and F8. 

Following the degree of polymerization trend, it can be deduced that fractions with lower DP were eluted first from the column and these represent monosaccharides and disaccharides. Later, the longer chain sugars, oligosaccharides, were eluted. This is because they were more strongly bound to the column by virtue of their charge and therefore required an eluent with higher ionic strength. 

### 2.2. FTIR Characterization

All employed fractions were analyzed using FTIR spectroscopy. The main functional groups present in the analyzed fractions are summarized in Table 2.

As clear from Table 2, S1 and V45 along with all column fractions show bands in the range 3500–3200 cm^−1^ corresponding to the stretching vibration of the hydroxyl group. They also exhibit bands in the range 1450–1350 cm^−1^, which can be ascribed to the stretching vibration of sulfate group (S=O bond) [22,23,24], in addition to the amide peaks at the wavelength range of 1670–1600 cm^−1^ [25].

Aromatic ester bands appeared at 1250–1310 cm^−1^ in F6, F7, F8, and S1 [26]. In addition, sulfoxide bands appeared in S1, V45, F1, F2, and F8 at 1055, 1058, 1059, 1075, and 1025 cm^−1^, respectively, and these could be attributed to the stretching vibration of S=O [27]. Only F8 and S1 showed bands corresponding to ester sulfate at 833 and 848 cm^−1^, respectively [28,29,30]. The ester sulfate bond is confirmed by the presence of both aromatic ester and sulfoxide bands in F8 and S1. As a result of hydrolysis, some of the ester sulfate bonds in S1 could have been broken and, thus, they appeared only in the highest molecular weight fraction (F8). Although the ester sulfate and aromatic ester bands did not appear in V45, these groups were probably present but in low concentrations that could not be detected by FTIR.

For fractions F3, F4, F5, F6, and F7, peaks appeared at wavenumbers of 1116, 1115, 1113, and 1111 cm^−1^, respectively. These correspond to the C=S stretching vibration of thiocarbonyl [23]. The absence of thiocarbonyl bands in S1 and V45 does not indicate the absence of thiocarbonyl groups since their concentrations are expected to be higher in the fractions than in S1 or V45 and, hence, were detectable in the fractions but not in S1 or V45.

Furthermore, the band pertaining to the O–H bending vibration of phenolic groups appeared at 1365 cm^−1^ in F3 only, while that corresponding to the thiol stretching vibration (S–H) appeared only in F8 at 2583 cm^−1^ [31,32]. In conclusion, all fractions along with S1 and V45 contain hydroxyl, sulfate, and amide groups. All fractions together with S1 and V45 and apart from (F3–F7) contain sulfoxide groups. F3–F7, however, possess thiocarbonyl groups instead. 

### 2.3. Antioxidant Activity

**Figure 2:**
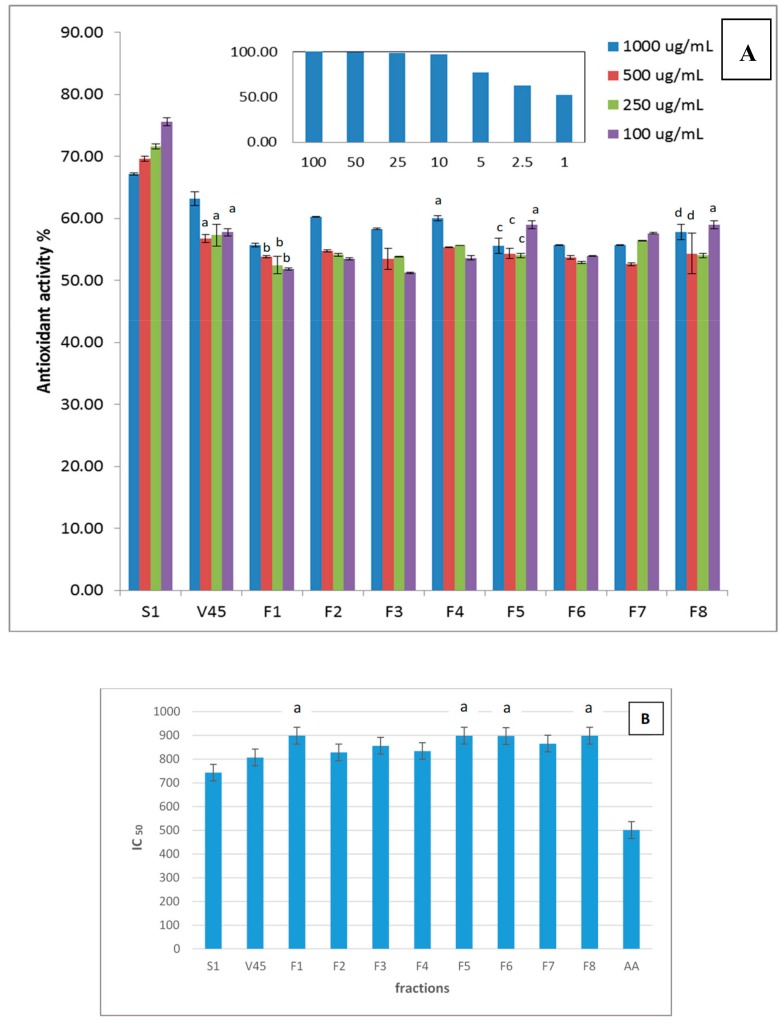
(**A**) Antioxidant activities of the column effluent fractions at different concentrations, along with those of the mother S1 extract and the enzymatically hydrolyzed extract V45. The figure inset shows the activity of ascorbic acid at different concentrations. Sets with the same letters have insignificantly different values at *p* = 0.05. (**B**) Relevant IC50 values.

The antioxidant activities of the column effluent fractions at different concentrations are depicted in Figure 2A, along with those of S1 and V45. The relevant IC50 values together with that of ascorbic acid (AA) are presented in Figure 2B.

As can be seen from the figure, the antioxidant activity is concentration dependent for S1 and all column fractions. It is also clear from the figure that at all concentrations, the activity of the mother fraction (S1) decreased with enzymatic hydrolysis. Furthermore, the anion exchange purification of the enzymatically hydrolyzed fraction did not improve the activity. The same finding was reported in a study on *Ganoderma atrum* mushrooms [19], and in another study performed on polysaccharides extracted from the brown seaweed *Saragasum pallidum*. In the latter, purification of the crude extracts onto DEAE column reduced their antioxidant activities [18].

Amongst the eight column effluent fractions (Figure 2), F2 and F4 are the only two fractions that showed their highest activities above 60% and these were achieved at 1000 μg/mL. These activities are slightly lower than that of V45 (63%) and are comparable to that of fraction F3 at the same concentration. The fractions F5 and F8 also showed comparable antioxidant activities but at lower concentrations.

The mechanism of DPPH scavenging activity is that of hydrogen transfer from the antioxidant to the DPP hydrazyl (radical) to convert it into DPP hydrazine. This is to avoid the presence of the active free radicals, which have the capability of degenerating the proteins, lipids, and DNA in human bodies or foods on the shelf, and, hence, lead to degenerative diseases [33]. The hydrogen transfer is suggested to take place through a possible reaction between the radical and amine or amide groups present in the antioxidant [34]. As deduced from the FTIR analysis, the amide group is shown in all fractions and, therefore, all of them have antioxidant activity. The highest in protein content are F2 and F4 and they are the only two fractions that showed antioxidant activity above the 60% so it could be attributed to the higher amount of protein, which indicates higher content of amide groups. However, there is no direct correlation between the antioxidant activity of the tested fractions and their protein content and this is because there are other functional groups that could be involved in the antioxidation reaction such as OH groups.

It can also be noted that the fractions with relatively higher antioxidant activities (F2–F5 and F8) all possess 1 < DP ≤ 3 except for fraction F8. The relatively high activity of F8 despite its high DP could be due to its possession of various functional groups probably at high concentrations. In addition, each of fractions F4, F5, and F8 along with S1 possess comparable carbohydrate and sulfate contents, as previously mentioned. This might suggest that the carbohydrate-to-sulfate ratio influences the antioxidant activity. Fractions F6 and F7, on the other hand, exhibited lower activities compared to the aforementioned fractions although they have a DP of 3. This could be owed to their possession of the highest sulfate contents in combination with the lowest protein contents amongst the investigated fractions. The negative effect of sulfates on the antioxidant activity was demonstrated in a study conducted on crude polysaccharides extracted from five different algal species. Therein, it was suggested that polysaccharides of the *Laminaria japonica* had the least antioxidant activity and the highest sulfate content due to the consumption of hydroxyl groups, a major player in the antioxidation process, with the sulfate groups [35]. Amongst all the column fractions, F1 with unity DP showed the lowest activity probably because it constitutes only monosaccharides. 

These findings suggest that various functional groups could be involved in the antioxidation process and these include hydroxyl, sulfate, and amide groups. Furthermore, DP plays an important role in the antioxidation capacity.

### 2.4. Antitumor Activity

As a preliminary investigation, the lethality percentage for each algal fraction on each of four cancer cell lines was determined to test its antitumor activity. Fractions with lethality of above 75% were considered promising. As can be seen in Table 3, four fractions S1, F4, F5, and F8 showed % lethality above 75. Fractions S1, F4, and F8 were lethal to HCT116, HePG2, and MCF7 cancer cells, respectively; whereas F5 was almost 100% lethal to both HePG2 and MCF7, and 80% lethal to A549 cancer cells. 

**Figure 3:**
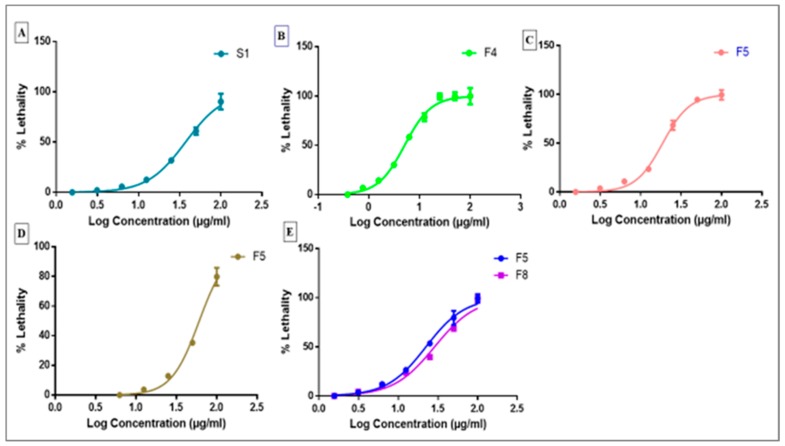
Dose–response curves for fractions S1 on HCT116 cells (**A**), F4 on HePG2 cells (**B**), F5 on HePG2 cells (**C**), F5 on A549 cells (**D**), and F5 and F8 on MCF7 cells (**E**). The dose–response curves were used to determine LC50, LC90, and Hill coefficient values.

Further investigations were performed on the fractions that showed 75% lethality or more. Dose–response curves (Figure 3) were generated for these fractions (S1, F4, F5, and F8) and used to determine LC50, LC0, and Hill coefficient values. Results obtained for both LC50 and LC90 are compiled in Table 4. LC50 and LC90 represent the concentration of the extract or fractions that led to death of the cancer cells by up to 50% and 90%, respectively.

**Figure 4:**
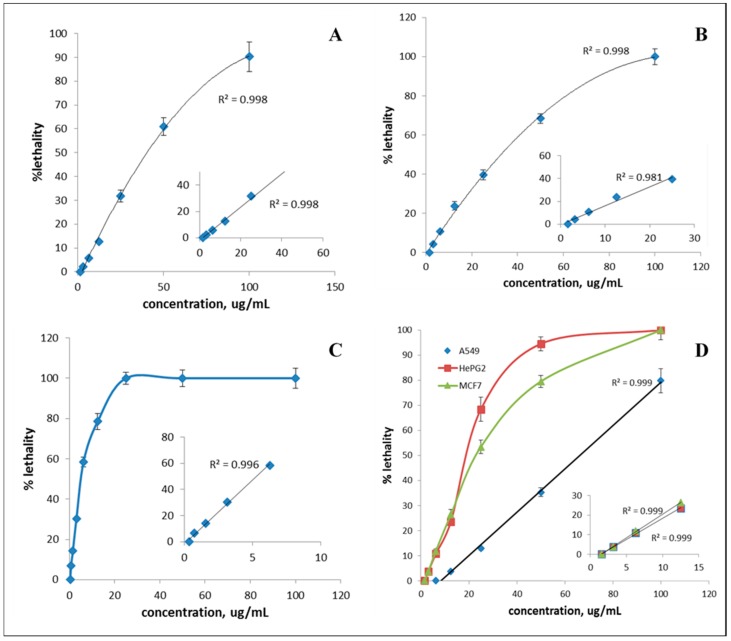
Evolution of % lethality with concentration for (**A**) S1 fraction on HCT116 colon cancer cells, (**B**) F8 fraction on MCF7 breast cancer cells, (**C**) F4 fraction on HePG2 human hepatocellular carcinoma cells, and (**D**) F5 fraction on A549, HePG2, and MCF7 cells. The linear parts of the curves are shown in the figure insets.

To examine the dependence of lethality on concentration, lethalities of the promising fractions at various concentrations were determined. The effect of concentration on the %lethality of S1 fraction on HCT116 cells is depicted in Figure 4A, where it can be observed that lethality is concentration dependent. Clearly, no lethality is detected for concentrations below or equal to 1.56 μg/mL; however, lethality increases with increasing concentration and its behavior follows a polynomial function trend with a correlation factor (R^2^) of 0.998. As for 1.56 ≤ concentrations ≤ 25 μg/mL, the lethality increases linearly with concentration (R^2^ = 0.998). Furthermore, the lethality-concentration profile for fraction F8 on breast cancer cells MCF7 is similar to that of S1 on HCT116 colon cancer cells (Figure 4B), and the correlation between lethality and concentration is linear for 1.56 ≤ concentrations ≤ 25 μg/mL (R^2^ = 0.981). F4, on the other hand, shows strong lethal effect on HePG2 human hepatocellular carcinoma cells as can be seen in Figure 4C, where no lethality is exhibited for concentrations below 0.38 μg/mL, then it increases linearly with concentration in the range (0.38–6.25) μg/mL (R^2^ = 0.996) and finally reaches a plateau at 25 μg/mL. This indicates that F4 has a strong antitumor activity with regard to HePG2 cells, even at relatively low concentrations.

Among the tested fractions, F5 showed a distinct behavior (Figure 4D), where it exhibited the best antitumor activity, with an almost 100% lethality on MCF7 and HePG2 at 100 and 60 μg/mL, respectively. This is in addition to 80% lethality on A549 at 100 μg/mL and the lethality-concentration relation was linear (R^2^ = 0.999) in this case. 

From the above, it can be concluded that S1, F4, F5, and F8 have potent antioxidant and antitumor activities probably since they are the only fractions that contain comparable amounts of carbohydrates and sulfates, i.e., carbohydrate-to-sulfate ratio of 1:1. Also notable is that F4 and F5 are both active on HePG2 cells and they are the only two among the tested fractions that share the same functional groups of hydroxyl, amide, sulfate, and thiocarbonyl, as well as the same approximate DP of 3. Fractions S1 and F8, on the other hand, showed antitumor activities on HCT116 and MCF7 cells, respectively; despite their high DP (high molecular weight). This could possibly be due to their carbohydrate-to-sulfate ratio of 1 and to their possession of a variety of functional groups. They both share hydroxyl, amide, sulfate, aromatic ester, sulfoxide, and ester sulfate groups, while F8 possesses additional thiol and phenolic groups. 

Interestingly, F6 and F7 showed no antitumor activity and relatively low antioxidant activity relative to F4 and F5, although they share the same DP of 3 with these two fractions. This could be attributed to their low protein content and hence their possession of less functional amide groups as compared to fractions 4 and 5. This is in addition to their highest sulfate contents amongst other fractions, which could have affected their antitumor activity as was the case with their antioxidant activity. Moreover, F6 and F7 share the same functional groups of hydroxyl, amide, sulfate, thiocarbonyl, and aromatic ester.

Another fraction that showed no antitumor activity, even though it comprises the same main functional groups present in fractions F4 and F5 (hydroxyl, amide, sulfate, and thiocarbonyl), is F3. This could be ascribed to its possession of unequal ratio of sulfate to carbohydrate content and probably due to its lower DP as compared to F4 and F5. However, F3 possesses an additional phenolic group that could have possibly played a role in imparting an antioxidant activity for this fraction that is comparable to those of F4 and F5. Antioxidant activity has been reported in the literature to be directly related to the phenolic content [21,36].

### 2.5. Hill Coefficient

**Figure 5:**
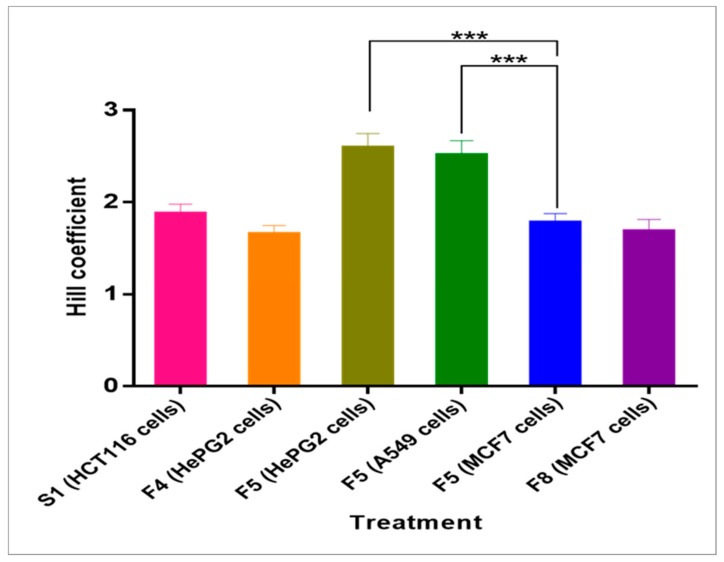
Hill coefficients for fractions that showed lethality ≥75%. The least Hill coefficient obtained was that of fraction F4 when tested on HepG2 cells while the greatest Hill coefficient was that of fraction F5 when tested on HePG2 cells. Fraction F5 showed significantly higher Hill coefficients (*** *p* < 0.001) when tested on both HePG2 and A549 cells than on MCF7 cells.

Hill coefficients (Table 5) were determined for fractions that showed lethality ≥75% and were all above 1. Hill coefficients can be used to give an idea of the degree of cooperativity among ligands as they bind to target macromolecules. Coefficients above, below, and equal to 1, respectively, indicate positive, negative, and non-cooperative ligand binding [37]. This suggests that the binding of one ligand promotes the binding of other ligands for fractions S1, F4, F5, and F8. The highest Hill coefficients were observed in treatments of fraction F5; treatment of F5 on HepG2 cells showed the greatest Hill coefficient (2.591 ± 0.155) followed by that on A549 (2.512 ± 0.156) and MCF7 (1.780 ± 0.097) cells, respectively (Figure 5). This is probably indicative of a higher degree of cooperativity in F5 treatments when compared to all others. An explanation for such a trend could most likely be due to the presence of multi-subunit "active components." Treatment of MCF7 cells with F5, however, resulted in a significantly lower Hill coefficient when compared to the treatment of either HePG2 or A549 cells. This suggests that ligands in the same fraction possibly interact with different sets of target molecules from one cell line to another. The above findings are in agreement with the antitumor results shown earlier, where F5 exhibited the highest percentage lethality on HePG2.

### 2.6. Sugar Analysis by HPLC

The sugar analysis (Table 6) was performed only on the mother algal extract together with F4, F5, and F8 since they exhibited the best antitumor activities among other fractions. They also showed potent antioxidant activities. 

As clear from the table, the most antitumor active fraction F5 is the highest in glucuronic acid while the second highest fraction in glucuronic acid is F4. Both of these fractions, as have been previously discussed, showed lethality for HePG2. In addition to their antitumor activity and as mentioned earlier, both F4 and F5 showed potent antioxidant activities that are, however, lower than that of S1. In a study conducted on the antioxidant activity of polysaccharides extracted from three different algal species, it was concluded that both the sulfate and glucuronic acid contents improve the activity. It was also reported that the sulfate groups are involved in the antitumor process by binding to the cationic protein on the cell surface avoiding its proliferation [13]. In the present work, F4 and F5 have higher glucuronic acid contents than S1, but lower sulfate contents. Hence, it can be inferred that the higher sulfate content of S1 could have led to its higher antioxidant activity, while the higher contents of glucuronic acid in F4 and F5 could be responsible for their higher antitumor activities. In addition to their highest glucuronic acid contents, F4 and F5 both have the lowest molar percentages of rhamnose. This is in contrary with S1 and F8 where rhamnose is the most abundant sugar unit. A distinguishable feature that is only pertinent to F5 is the possession of comparable contents of rhamnose and glucuronic acid on the one hand, and the same for glucose and arabinose on the other hand.

In addition to sugar composition, the complexity of the polysaccharide in terms of the variety of sugar units constituting its chain is another important influencing factor. Previous studies reported that better lung cancer A549 antitumor activity was exhibited with the more complex polysaccharides that contained different kinds of monosaccharides. The more complex ones comprised eight kinds of monosaccharides as opposed to other samples that contained three and five types only [38]. Sugar analysis in the present study revealed that F5 contains the five tested monomers while F4 and F8 comprise only four monomers and this could explain the high antitumor activity exhibited by F5 on three types of cells rather than only one type as is the case with other fractions. However, the algal extract S1 contains five monomers but is active on one cell line only, which suggests that the molecular weight along with the complexity of the molecule should be taken into consideration. F5 showed better antitumor performance than S1 probably due to its lower DP, as previous studies showed that antitumor activity is enhanced with lower molecular weights [18,39]. In addition, F5 comprises a thiocarbonyl group while S1 possesses an ester sulfate group instead [38]. Most importantly, the superior activity of F5 relative to other fractions could be ascribed to a feature that is characteristic only of this fraction. This feature is having comparable contents of proteins, sulfates, and carbohydrates, in addition to having comparable contents of rhamnose and glucuronic acid, and the same for glucose and arabinose.

To sum up, S1 along with F4, F5, and F8 showed potent antioxidant as well as antitumor activities due to a number of factors. The ratio of carbohydrate to sulfate content could be majorly responsible for this activity since they all possess a carbohydrate to sulfate ratio of about 1:1. Furthermore, other factors could affect the biological activity of a molecule as was shown earlier; namely, DP, type of characteristic functional groups like sulfate, hydroxyl, and amide, as well as polymer complexity and monomer percentage. Additional reported factors include, but are not limited to, the arrangement of functional groups and monomer distribution and branching [18,40]. It remains to be determined whether the SPs of the crude extract S1 and the isolated fractions F4, F5, and F8 of the green algae effect on cell proliferation was attributed to genetic or epigenetic alterations in the cellular milieu. Jose and colleagues [41] reported that SPs isolated from the brown marine algae *Padina tetrastromatica* reduced the expression levels of genes known to promote cell growth, angiogenesis, and metastasis. Moreover, anti-cancer therapeutic agents such as oxaliplatin were proven to be associated with epigenetic modulations as depicted by the miRNA expression profile in HCT116 in response to the treatment [42]. It is also possible that the SPs are contributing to the modification of the aberrant glycosylation on the surface of cancer cells tumor-associated carbohydrates [43]. As stated and referenced earlier, the extracts properties and biological activities, to a great extent, depend on the number and the location of their sulfate groups [44].

## 3. Materials and Methods 

### 3.1. Extraction of SPs from Algae

Green *Ulva lactuca* algae were collected in summer time from the Mediterranean Sea in Egypt, specifically Alexandria in Abou Kir region (N 31°19′ E 30°03′). Algae were washed with tap water followed by deionized water, then dried and ground to start extraction.

Conventional acid hydrolysis was conducted by stirring the algae for 2 h in water at 80 °C and pH 5 adjusted using 1 N HCl. This was followed by filtration of the extract and then the extract filtrate was kept in a dialysis bag for 48 h and at pH 7 adjusted using sodium carbonate saturated solution [45,46]. Finally, the extract was centrifuged (Heraeus-Christ, GMBH336 Osteode Ma Harz No.39189) at 12,000× *g* for 15 min at −10 °C to get rid of any undissolved molecules. The relatively high molecular weight SPs (S1) was precipitated using ethyl alcohol with a ratio of 4:1 alcohol to extract. They were then separated from lower molecular weight ones (supernatant) using centrifugation, and finally dried and collected for further investigations. 

### 3.2. Enzymatic Hydrolysis and Anion-Exchange Chromatography

The fraction S1 was hydrolyzed into smaller molecules using an enzyme mixture called viscozyme L (a multi-enzyme complex containing arabinase, cellulase, β-glucanase, hemicellulase, and xylanase), purchased from Sigma-Aldrich (St. Louis, MO, USA). The presence of multiple enzymes would potentially produce several sugar chains with different lengths and various characteristics. First, S1 was dissolved in sodium acetate buffer (pH 5.6) where 0.25 g of which were dissolved in 10 mL buffer. A volume of 225 µL of the enzyme was added to S1, then left in a rotating shaker at 37 °C and 50 rpm for 45 min. The product (V45) was frozen for 30 minutes to deactivate the enzyme, then centrifuged to collect the supernatant, which was dried.

The hydrolysate (V45) was further purified using diethylaminoethyl (DEAE) cellulose anion-exchange column chromatography. The column (Whatman International Ltd., Maidstone, UK) was packed with 50 g of DEAE cellulose suspended in 0.1 M of 98% ammonium acetate (Sigma-Aldrich, St. Louis, MO, USA) buffer (pH 5.6). Column dimensions were 16.1 cm length and 1.3 cm in diameter. Before sample application, the column was first equilibrated with 3 column volumes of acetate buffer. Then, 1 μg/L of V45 was prepared in the same buffer, filtered, then added to the column. Afterwards, the column was eluted at a flow rate of 2.5 mL/min with 100 mL of ammonium carbonate (Sigma-Aldrich, St. Louis, MO, USA) in a gradient manner from 0 to 1 M (0 M, 0.1 M, 0.2 M, 0.4 M, 0.6 M, 0.8 M, 1 M) in addition to a final saturated solution. Eight fractions were thus collected (F1–F8). All fractions were dried and used for further chemical and biological investigations.

### 3.3. Chemical Tests

#### 3.3.1. Determination of Carbohydrate, Protein, and Sulfate Contents

Total carbohydrate content of the water-soluble sulfated polysaccharide (S1) extracted from *Ulva lactuca* together with the enzymatically extracted fraction V45 and the isolated fractions (F1–F8) from the column were determined to adopt the phenol-sulfuric acid assay [47] with some modifications [21] and using glucose as a standard. 

The protein contents of the investigated polysaccharide preparations were determined adopting the method of Lowry et al. [22] and using bovine albumin as a standard. 

The sulfate content, on the other hand, was determined after cleavage of the polysaccharide molecule using the method of Larsen et al. [48]. Sulfate contents of the hydrolysates were determined to adopt the barium chloride turbidimetric assay of Garrido [49], with some modifications [21]. The amount of sulfate was estimated based on the anhydrous sodium sulfate standard calibration curve.

#### 3.3.2. Determination of Degree of Polymerization (DP)

DP was determined according to the method of Timell [50] for the following samples (V45, F1–F8), while S1 being the high molecular weight polysaccharide was excluded from the determination. For reduction, 1 mL (100 µg/mL) of each sample was mixed with 0.5 mL sodium borohydride solution (1%) and reduction was allowed to proceed for 1 h in the dark at room temperature. Another similar set of samples were prepared simultaneously with the addition of 0.5 mL of 2N sulfuric acid. To both sets, 1 mL of aqueous phenol (3%) was first added, followed by 5.0 mL of concentrated sulfuric acid. After thorough mixing, the solutions were allowed to stand for 10 minutes at room temperature, then the test tubes were left to cool in a water bath for another 25 minutes. Absorbance measurements were carried out at 480 nm and the average of each set of the determinations was used for calculating *DP* of the investigated samples using the following relation

(1) $$DP=\;\frac{Q}{Q-1}$$

where *Q* is the absorbance ratio (*A*_1_/*A*_2_), and *A*_1_ and *A*_2_ are the respective absorbances of SPs samples before and after reduction.

#### 3.3.3. Determination of Sugar Content

Samples were analyzed for mono- and disaccharides by high-performance liquid chromatography (HPLC) according to AOAC [51]. Sugars were extracted using double distilled water for 3 h; the extract was passed through C18 Sep-Pakcartridge, refrigerated, and stored for further analysis. Standard solutions of glucose, glucuronic acid, rhamnose, galactose, and xylose sugars were prepared by diluting each analyzed sugar with deionized water. These sugars were chosen for analysis as they are the most abundant sugars in *Ulva lactuca*.

For sugar content analysis, samples were filtered through a 0.45 µm membrane to be analyzed onto an HPLC, Shimadzu Class-VPV 5.03 (Shimadzu Corporation, Kyoto, Japan) equipped with refractive index RID-10A Shimadzu detector, LC-16ADVP binary pump, DCou-14 A degasser, Shodex PL Hi-Plex Pb column (Sc 1011 No. H706081) Guard column Sc-Lc Shodex, and a heater set at 80 °C. Separation and quantitation were carried out with deionized water as the mobile phase. Injection volume for each standard and sample was 20 µL.

### 3.4. Fourier Transform Spectral Analysis

Fourier transform infrared spectroscopy was performed for S1, V45, and all 8 column fractions. Samples were mixed with potassium bromide in the form of 1-mm pellets and analyzed in a TGA/FT-IR Nicolet 380 spectrometer for a range of wavenumbers between 500 and 4000 cm^−1^. 

### 3.5. Biological Tests

#### 3.5.1. Antioxidant Activity

Following Blois [52] with minor modifications, 1 M of 2,2-diphenyl-1-picrylhydrazyl (DPPH) solution (95% methanol) along with samples of different concentrations 50, 100, 250, 500, and 1000 μg/mL were prepared. A volume of 2 mL of the sample was mixed with 2 mL of DPPH solution for 1 h in the dark. A colorimetric assay was performed by measuring absorbance at 517 nm to detect the decolorization. A methanol solution (95%) was used as a blank, while standards of ascorbic acid were used as references. The scavenging activity was calculated according to the following equation [21]

(2) $$Scavenging\;rate\%=\;\frac{\left[A_{0}-(A_{2}-A_{1}\right)]}{A_{0}}\times 100$$

where *A*_0_ is the absorbance of the sample-free DPPH solution, *A*_1_ is the absorbance of the tested samples in absence of DPPH, and *A*_2_ is the absorbance of the tested samples in presence of DPPH. IC50 was estimated as the sample concentration (μg/mL) required to scavenge 50% of the DPPH.

#### 3.5.2. Antitumor Activity

The antitumor cell test depends basically on the cytotoxic effect of each of the samples on the cancer cells. In this test, cell viability was measured, as per Mosmann assay [53,54], by reduction of yellow MTT (3-(4,5-dimethylthiazol-2-yl)-2,5-diphenyltetrazolium bromide) to purple formazan via mitochondria.

All the following procedures were done in a sterile area using a Laminar flow cabinet biosafety class II level (Baker, SG403INT, Sanford, ME, USA). Cells were suspended in Roswell Park Memorial Institute RPMI 1640 medium for HePG2, MCF7, and HCT116, and in Dulbecco’s Modified Eagle Medium DMEM for A549. The media were supplemented with 1% antibiotic-antimycotic mixture to prevent any bacterial or fungal contamination (10,000 U/mL Potassium Penicillin, 10,000 µg/mL Streptomycin Sulfate, and 25 µg/mL Amphotericin B), 1% L-glutamine, and 10% fetal bovine serum to help in the growth of cells and the mixture was kept at 37 °C under 5% CO_2_.

Cell culture was conducted in batch for 10 days, and cells were afterwards seeded at a concentration of 10 × 10^3^ cells/well in fresh complete growth medium placed in 96-well microtiter plastic plates. The plates were left for 24 h in a water-jacketed carbon dioxide incubator (Sheldon, TC2323, Cornelius, OR, USA) at 37 °C and under 5% CO_2_. Media was aspirated, and fresh medium (without serum) was added. Cells were incubated either alone to determine the negative control, or with different concentrations of the sample to yield final concentrations of 100, 50, 25, 12.5, 6.25, 3.125, 1.56, and 0.78 μg/mL. After 48 h of incubation, the medium was aspirated and 40 μL of 2.5 μg/mL of MTT salt were added to each cell and incubated for further 4 h at 37 °C under 5% CO_2_. To stop the reaction and dissolve the formed crystals, 200 μL of 10% Sodium Dodecyl Sulfate (SDS) in deionized water was added to each well and incubated overnight at 37 °C. Doxorubicin was used as a positive control at 100 µg/mL since it is a known cytotoxic natural agent, which gives 100% lethality under the same conditions as per Thabrew et al. [55,56]. 

The absorbance was then measured using a microplate multi-well reader (Bio-Rad Laboratories Inc., model 3350, Hercules, CA, USA) at 595 nm and a reference wavelength of 620 nm. A statistical significance was tested between samples and negative control (cells with vehicle) using independent *t*-test by SPSS 11 program. Dimethyl sulfoxide (DMSO) was the vehicle used for the dissolution of algae extract and its fractions, and its final concentration on the cells was less than 0.2% [55,56]. The percentage of change in viability was calculated according to the formula [56] where extract reading was normalized against the negative control:

(3) $$\%\;change\;in\;viability=\left[\frac{reading\;of\;extract}{reading\;of\;negative\;control}-1\right]\times 100.$$

The samples that showed 75% or more lethality at 100 ppm were further investigated for lower concentrations. The cancer cells used were all from the American type culture collection ATCC (USA) and were four types (HCT116, colon cell line; A549, lung carcinoma cell line; HePG2, human hepatocellular carcinoma cell line; MCF7, breast carcinoma). Each sample was tested for its effect on each of the four types.

#### 3.5.3. LC50, LC90, and Hill Coefficient 

Results obtained from the MTT assay were used to determine both LC50 and LC90 values for fractions that showed lethality greater than 75%. Using non-linear regression analysis, dose–response curves were fitted in GraphPad Prism version 6.0 for Windows, GraphPad Software, La Jolla California USA, www.graphpad.com. The following equations were used for curve fitting:

Y = Bottom + (Top − Bottom)/(1 + 10^((LogEC50 − X) × HillSlope)(4)

logEC50 = logECF − (1/HillSlope) × log(F/(100 − F))(5)

where X is log of concentration of treatment, Y is % Lethality, Top is Top plateau of regression curve in units of *Y* axis, Bottom is Bottom plateau of regression curve in units of *Y*-axis, and F is 90.

Equations (4) and (5) were, respectively, used to determine LC50 and LC90 values from the fitted curves; both EC50 and ECF in the equations are, respectively, analogous to LC50 and LC90. Furthermore, values for ‘Top’ and ‘Bottom’ in Equation (4) were constrained to 100 and 0, respectively.

LC50 values were defined as the concentration of the treatment required to bring percentage lethality to a point halfway between the top and bottom plateaus of the dose–response curves while LC90 values were defined as the concentration of the treatment required to bring percentage lethality to 90%. Finally, the Hill slope was defined as the steepness of a given dose response curve and was considered to be equivalent to the Hill coefficient [57].

## 4. Conclusions

To conclude, the four effluent fractions S1, F4, F5, and F8 that possessed comparable carbohydrate to sulfate contents showed potent antitumor activities. More specifically, F5 was highly active on three cell lines, while each of S1, F4, and F8 were active on only one cell line. This remarkable feature of F5 that was not observed in the other fractions could be attributed to its comparable contents of proteins, carbohydrates, and sulfates, as well as comparable contents of rhamnose and glucuronic acid, and the same for glucose and arabinose. The highest Hill coefficient of F5 relative to the other promising fractions indicates a higher degree of cooperativity in ligand binding. Other influencing factors could be its relatively low DP, as well as its possession of a variety of sugar units and functional groups.

Findings of this study suggest that the antioxidant and antitumor activities are mainly influenced by the carbohydrate to sulfate ratio, DP, protein content, sugar composition, and variation as well as type and variety of functional groups.

The proposed process combining enzymatic hydrolysis and ion-exchange chromatography could potentially be used in food and pharmaceutical industries as a post extraction step to produce compounds with better bioactivities than the polysaccharide extracts. The process is relatively facile with few number of steps and is green with no solvent requirements. In addition, this study elucidated some of the important factors that could influence the bioactivity. Major factors that, to our best knowledge, have not been reported elsewhere are the carbohydrate to protein to sulfate composition and the sugar composition. In view of these influential factors, tailor-made oligosaccharides could be synthesized and designed as food supplements. This study should also be beneficial for further follow-up studies on structure–activity relationships or structure determination. Building on this study, future work should focus on investigating the specific bioactives through chemical profiling and probably further purifying only the active fractions that have the overall characteristics identified in this work.

## Figures and Tables

**Table 1:** Protein, carbohydrate, and sulfate contents together with the degree of polymerization for the parent algal extract, the hydrolysate, and the column effluent fractions.

Fraction	Protein (%)	Carbohydrate (%)	Sulfate (%)	DP
F1	11.3 ± 0.9	13.8 ± 1.1	1.20 ± 0.10	1
F2	18.8 ± 0.8	29.3 ± 2.1	0.625 ± 0.040	1.3~1
F3	7.50 ± 0.60	3.70 ± 0.26	9.06 ± 0.70	1.2~1
F4	26.9 ± 1.9	11.0 ± 0.9	11.6 ± 0.9	2.5~3
F5	10.6 ± 0.9	14.9 ± 1.1	15.5 ± 1.1	3
F6	3.10 ± 0.05	15.0 ±1.3	38.0 ± 2.9	3
F7	0.200 ± 0.001	25.0 ± 2.0	32.0 ± 2.7	3.3~3
F8	0.100 ± 0.007	16.1 ± 2.1	12.00 ± 1.90	4.5~5
V45	13.1 ± 1.1	29.1 ± 2.1	4.10 ± 0.30	6
S1	5.20 ± 0.31	36.2 ± 3.5	32.3 ± 3.1	___

* Values except DP are expressed as mean ± SD.

**Table 2:** Summary of the FTIR spectral analysis.

Functional Group	Wavelength Range (cm^−1^)	Bond	S1	V45	F1	F2	F3	F4	F5	F6	F7	F8
Hydroxyl	3500–3200	O-H stretch	√	√	√	√	√	√	√	√	√	√
Amide	1670–1600	C=O stretch	√	√	√	√	√	√	√	√	√	√
Sulfate	1450–1350	S=O stretch	√	√	√	√	√	√	√	√	√	√
Sulfoxide	1060–1030	S=O stretch	√	√	√	√	X	X	X	X	X	√
Ester sulfate	805–900	C-O-S stretch	√	X	X	X	X	X	X	X	X	√
Aromatic ester	1310–1250	C=O stretch	√	X	X	X	X	X	X	√	√	√
Thiocarbonyl	1060–1200(~1110)	C=S stretch	X	X	X	X	√	√	√	√	√	X

√: present, X: absent.

**Table 3:** Lethality percentage for the algal extract and column fractions at 100 ppm, as tested on four cancer cell lines.

Fraction	HePG2	HCT116	A549	MCF7
F1	12.6 ± 1.1	0.0 ± 0.1	65.3 ± 4.1	62.8 ± 3.5
F2	20.1 ± 2.5	4.0 ± 0.3	20.8 ± 2.0	0.0 ± 0.1
F3	11.2 ± 1.3	14.8 ± 1.4	4.1 ± 0.2	31.4 ± 2.8
F4	99.9 ± 8.2	32.8 ± 2.9	21.1 ± 1.6	58.2 ± 5.0
F5	99.5 ± 5.0	41.1 ± 2.8	79.8 ± 6.0	99.0 ± 4.3
F6	46.8 ± 3.8	−243 ± 14.0	−341 ± 20.5	−116 ± 10.2
F7	29.7 ± 1.9	66.7 ± 7.0	14.7 ± 11.3	49.3 ± 3.9
F8	32.2 ± 3.1	51.8 ± 4.8	19.6 ± 1.5	99.2 ± 4.1
V45	40.4 ± 3.6	43.6 ± 3.7	−128 ± 11.5	0.0 ± 0.0
S1	54.1 ± 4.8	90.2 ± 7.8	−210 ± 15.0	12.9 ± 1.8

**Table 4:** LC50 and LC90 for the fractions that showed lethality ≥75%.

Fraction	Cancer Cells	LC50 (µg/mL)	LC90 (µg/mL)
S1	HCT116	37.28 ± 1.032	120.3 ± 1.075
F4	HePG2	5.063 ± 1.039	19.12 ± 1.086
F5	HePG2	18.66 ± 1.027	43.57 ± 1.059
F5	A549	60.68 ± 1.026	145.5 ± 1.064
F5	MCF7	22.32 ± 1.035	76.69 ± 1.078
F8	MCF7	28.65 ± 0.022	105.6 ± 1.120

**Table 5:** Hill coefficients for fractions that showed lethality ≥75%.

Fraction (Cell Line)	Hill Coefficient
S1 (HCT116)	1.876 ± 0.102
F4 (HePG2)	1.654 ± 0.092
F5 (HePG2)	2.591 ± 0.155
F5 (A549)	2.512 ± 0.156
F5 (MCF7)	1.780 ± 0.097
F8 (MCF7)	1.684 ± 0.129

**Table 6:** Molar sugar content, as detected by HPLC, of the biologically active fractions along with the hydrolysate and the mother algal extract.

	Glucose	Arabinose	Xylose	Rhamnose	Glucuronic Acid
S1	23.8 ± 1.9	19.5 ± 1.1	5.30 ± 0.05	36.5 ± 2.0	14.9 ± 1.0
F4	23.2 ± 1.8	44.8 ± 3.1	14.3 ± 1.0	___	17.8 ± 1.5
F5	18.0 ± 1.3	16.6 ± 1.2	9.70 ± 0.50	30.0 ± 2.5	25.8 ± 2.0
F8	9.50 ± 0.03	___	12.4 ± 0.9	69.1 ± 4.3	9.00 ± 0.06

## References

[1] Lahaye M. (1998). NMR spectroscopic characterisation of oligosaccharides from two *Ulva rigida* ulvan samples (Ulvales, Chlorophyta) degraded by a lyase. Carb. Res..

[2] Jiao G., Yu G., Zhang J., Ewart H. (2011). Chemical structures and bioactivities of sulfated polysaccharides from marine algae. Mar. Drugs.

[3] Qi H., Zhao T., Zhang Q., Li Z., Zhao Z., Xing R. (2005). Antioxidant activity of different molecular weight sulfated polyssacharides from *Ulva pertusa Kjellum* (Chlorophyta). J. Appl. Phycol..

[4] Abd El-Baky H.H., El Baz F.K., El-Baroty G.S. (2008). Evaluation of marine alga *Ulva lactuca* L. as a source of natural preservative ingredient. Electron. J. Environ. Agric. Food Chem..

[5] Meenakshi S., Manicka D.G., Tamil S., Arumugam M., Balasubramanian T. (2009). Total flavanoid and in vitro antioxidant activity of two seaweeds of Rameshwaram Coast. Glob. J. Pharmacol..

[6] Kokabi M., Yousefzadi M., Ahmadi A., Feghhi M., Amin K.M. (2013). Antioxidant activity of extracts of selected algae from the Persian Gulf, Iran. J. Persian Gulf.

[7] Farasat M., Khavari-nejad R.A., Nabavi S.M.B., Namjooya F. (2014). Antioxidant activity, total phenolics and flavonoid and contents of some edible seaweeds from Northern Coasts of Persian Gulf Iran. J. Pharm. Res..

[8] Khairy H., El-Sheikh M. (2015). Antioxidant activity and mineral composition of three Mediterranean common seaweeds from Abu-Qir Bay, Egypt. Saudi J. Biol. Sci..

[9] El-Sayed M.H., Fleita D., Rifaat D., Essa H., Alexandru M.G., Alina M.H. (2018). Ingredients Extraction by Physicochemical Methods in Food.

[10] Fan S., Zhang J., Nie W., Zhou W., Jin L., Chen X., Lu J. (2017). Antitumor effects of polysaccharide from Sargassum fusiforme against human hepatocellular carcinoma HepG2 cells. Food Chem. Toxicol..

[11] Peasura N., Laohakunjit N., Kerdchoechuena O., Vongsawasdib P., Chao K.L. (2016). Assessment of biochemical and immunomodulatory activity of sulfated polysaccharides from *Ulva intestinalis*. Int. J. Biol. Macromol..

[12] Boopathy N.S., Kathiresan K., Dominguez H. (2013). Anticancer agents derived from marine algae. Functional Ingredients from Algae for Foods and Nutraceuticals.

[13] Shao P., Chen X., Sun P. (2013). *In vitro* antioxidant and antitumor activities of different sulfated polysaccharides isolated from three algae. Int. J. Biol. Macromol..

[14] Kaeffer B., Bénard C., Lahaye M., Blottière H.M., Cherbut C. (1999). Biological properties of ulvan, a new source of green seaweed sulfated polysaccharides, on cultured normal and cancerous colonic epithelial cells. Planta Medica.

[15] Ahmed O., Ahmed R. (2014). Anti-Proliferative and apoptotic efficacies of ulvan polysaccharides against different types of carcinoma cells in vitro and in vivo. J. Cancer Sci. Ther..

[16] Thanh T.T.T., Quacha T.M.T., Nguyenb T.N., Luonga D.V., Buic M.L., Tran T.T.V. (2016). Structure and cytotoxic activity of ulvan extracted from green seaweed *Ulva lactuca*. Int. J. Biol. Macromol..

[17] Sun L., Wang C., Shi Q., Ma C. (2009). Preparation of different molecular weight polysaccharides from *Prophyridium cruentum* and their antioxidant activities. Int. J. Biol. Macromol..

[18] Ye H., Wang K., Zhou C.J., Zeng X. (2008). Purification, antitumor and antioxidant activities in vitro of polysaccharides from the brown seaweed *Sargassum pallidum*. Food Chem..

[19] Chen Y., Xie M., Nie S., Li C., Wang Y. (2008). Purification, composition analysis and antioxidant activity of a polysaccharide from the fruiting bodies of *Ganoderma atrum*. Food Chem..

[20] Di T., Chen G., Sun Y., Ou S., Zeng X., Ye H. (2017). Antioxidant and immunostimulating activities in vitro of sulfated polysaccharides isolated from *Gracilaria rubra*. J. Funct. Foods.

[21] Fleita D., El-Sayed M., Rifaat D. (2015). Evaluation of the antioxidant activity of enzymatically-hydrolysed sulfated polysaccharides extracted from red algae; *Pterocladia capillacea*. LWT-Food Sci. Technol..

[22] Lowry O.H., Rosenbrough N.J., Farr A.L., Randall R.J. (1951). Protein measurement with the Folin phenol reagent. J. Biol. Chem..

[23] Agardh J., Vasquez R., Donnie J., Ramos A., Bernal S. (2012). Chemopreventive properties of sulfated polysaccharide extracts from *Saragasum siliquosum*. Int. J. Pharm. Biol. Sci..

[24] Kannan S. (2014). FT-IR and EDS analysis of the seaweeds *Sargassum wightii* (brown algae) and *Gracilaria corticata* (red algae). Int. J. Curr. Microbiol. Appl. Sci..

[25] Radhika D., Mohaideen A. (2015). Fourier transform infrared analysis of *Ulva lactuca* and *Gracilaria corticata* and their effect on antibacterial activity. Asian J. Pharm. Clin. Res..

[26] Hortin G.L., Goldberger G.A., Burtis C.A., Ashwood E.R., Bruns D.E. (2012). Chromatography and extraction. Tietz Textbook of Clinical Chemistry and Molecular Diagnosis.

[27] Ji A., Yao Y., Che O., Wang B., Sun L., Li X., Xu F. (2011). Isolation and characterization of sulfated polysaccharide from the *Sargassum pallidum* (Turn.) C. Ag. and its sedative/hypnotic activity. J. Med. Plants Res..

[28] Tian H., Yin X., Zeng Q., Zhu L., Chen J. (2015). Isolation, structure, and surfactant properties of polysaccharides from *Ulva lactuca* L. from South China Sea. Int. J. Biol. Macromol..

[29] Pereira L., Amado A., Critchely A., Velde F., Claro P. (2009). Identification of selected seaweed polysaccharides (phycocolloids) by vibrational spectroscopy (FTIR-ATR and FT-Raman). Food Hydrocoll..

[30] Ordonez F., Ruperez P. (2011). FTIR-ATR spectroscopy as a tool for polysaccharide identification in edible brown and red seaweeds. Food Hydrocoll..

[31] Tingaut P., Hauert R., Zimmermann T. (2011). Highly efficient and straight forward functionalization of cellulose films with thiol-ene. J. Mater. Chem..

[32] Guan X., Shang C., Chen G. (2006). ATR-FTIR investigation of the role of phenolic groups in the interaction of some NOM model compounds with aluminum hydroxide. Chemosphere.

[33] Rajani S., Gokilai M., Jency P., Brindha P., Sujathai R. (2011). Antioxidant and phytochemical properties of *Aegle marmelos* fruit pulp. Int. J. Curr. Pharm. Res..

[34] Chen S., Tsai M., Huang J., Chen R. (2009). *In vitro* antioxidant activities of low-molecular-weight polysaccharides with various functional groups. J. Agri. Food Chem..

[35] Zhang Z., Wang F., Wang X., Liu X., Hou Y., Zhang Q. (2010). Extraction of the polysaccharides from five algae and their potential antioxidant activity in vitro. Carbohyd. Polym..

[36] Thetsrimuang C., Khammuang S., Sarnthima R. (2011). Antioxidant activity of crude polysaccharides from edible fresh and dry mushroom fruiting bodies of *Lentinus* sp. strain RJ-2. Int. J. Pharmacol..

[37] Weiss J.N. (1997). The Hill equation revisited: Uses and misuses. FASEB J..

[38] He R., Zhao Y., Zhao R., Sun P. (2015). Antioxidant and antitumor activities in vitro of polysaccharides from *E. sipunculoides*. Int. J. Biol. Macromol..

[39] Hao W., Wang X., Li H., Wang S., Chen T., Yuan Z., Tang Y. (2014). Isolation and characterization of polysaccharides with the antitumor activity from *Tuber* fruiting bodies and fermentation system. Appl. Microbiol. Biotechnol..

[40] Dore C., Alves M., Santos M., Cruz A., Camara R., Castro A., Alves L., Nader L., Leite E. (2013). Antiangiogenic activity and direct antitumor effect from a sulfated polysaccharide isolated from seaweed. Microvasc. Res..

[41] Jose G.M., Kurup G.M. (2017). Sulfated polysaccharides from *Padina tetrastromatica* arrest cell cycle, prevent metastasis and down regulate angiogenic mediators in HeLa cells. Bioact. Carbohydr. Diet. Fibre.

[42] Evert J., Pathak S., Sun X.F., Zhang H. (2018). A Study on Effect of Oxaliplatin in MicroRNA Expression in Human Colon Cancer. J. Cancer.

[43] Soliman C., Yuriev E., Ramsland P.A. (2017). Antibody recognition of aberrant glycosylation on the surface of cancer cells. Curr. Opin. Struct. Biol..

[44] Khotimchenko Y.S. (2010). Antitumor Properties of Non starch Polysaccharides: Fucoidans and Chitosans. Russ. J. Mar. Biol..

[45] Hussien M.M. (1977). Biochemical studies on the Egyptian marine algae. The water soluble polysaccharides of *Ulva lactuca*. Pak. J. Biochem. Mol. Biol..

[46] Essa H., Fleita D., Rifaat D., Samy S., El-Sayed M.H. (2018). Towards optimizing the conventional and ultrasonic-assisted extraction of sulphated polysaccharides from Marine Algae. IOP Conf. Ser. Mater. Sci. Eng..

[47] Dubois M., Gillis K.A., Hamilton J.K., Rebers P.A., Smith F. (1956). Colorimetric methods for determination of sugars and related substances. Anal. Chem..

[48] Larsen B., Haug A., Painter J.T.J. (1966). Sulfated polysaccharides in brown algae. Isolation and preliminary characterization of three sulfated polysaccharides from *Ascophyllum nodosum*. Acta Chem. Scand..

[49] Garrido M.L. (1964). Determination of sulphur in “plant material”. Analyst.

[50] Timell T.E. (1960). Determination of the degree of polymerization of reducing pentose and hexose oligosaccharides. Svensk Papperstidning.

[51] AOAC (1997). Official Methods of Analysis.

[52] Blois M.S. (1958). Antioxidant determination by the use of a stable free radical. Nature.

[53] Mosmann T. (1983). Rapid colourimetric assays for cellular growth and survival, Application to proliferation and cytotoxicity assays. J. Immunol. Methods.

[54] Sreekumar B., Bansal A., Ray A., Rao A., Mishra A. (2003). A rapid MTT colourimetric assay to assess the proliferative index of two Indian strains of *Theileria annulata*. Vet. Parasitol..

[55] Septisetyani E., Ningrum R., Romadhani Y., Wisnuwardhani P., Santoso A. (2014). Optimization of sodium dodecyl sulfate as a formazan solvent and comparison of 3- (4, -5 dimethylthiazo-2-yl)- 2.5 diphenyl tetrazolim bromide (MTT) assay with WST- 1 assay in MCF7 cells. Indones. J. Pharm..

[56] Thabrew M.I., Hughes R.D., McFarlane I.G. (1997). Screening of hepatoprotective plant components using a HepG2 cell cytotoxicity assay. J. Pharm. Pharmacol..

[57] Graphpad Software Inc. (2015). GraphPad Curve Fitting Guide. https://www.graphpad.com/guides/prism/6/curve-fitting/index.htm?reg_dr_inhibit_variable.htm.

